# Oncogenic Signaling Alters Cell Shape and Mechanics to Facilitate Cell Division under Confinement

**DOI:** 10.1016/j.devcel.2020.01.004

**Published:** 2020-03-09

**Authors:** Helen K. Matthews, Sushila Ganguli, Katarzyna Plak, Anna V. Taubenberger, Zaw Win, Max Williamson, Matthieu Piel, Jochen Guck, Buzz Baum

**Affiliations:** 1 MRC Laboratory for Molecular Cell Biology, University College London, Gower Street, London WC1E 6BT, UK; 2 The Francis Crick Institute, 1 Midland Road, London NW1 1AT, UK; 3 Biotechnology Center, Technische Universität Dresden, Tatzberg 47/49, 01307 Dresden, Germany; 4 Institut Curie and Institut Pierre Gilles de Gennes, PSL Research University, CNRS, UMR 144, Paris, France; 5 Max Planck Institute for the Science of Light & Max-Planck-Zentrum für Physik und Medizin, Staudtstraße 2, 91058 Erlangen, Germany; 6 Institute for the Physics of Living Systems, University College London, London WC1E 6BT, UK

**Keywords:** mitotic rounding, mitosis, actin, Ras, MAPK signaling, MEK, ERK, cell mechanics, cancer, cell confinement

## Abstract

To divide in a tissue, both normal and cancer cells become spherical and mechanically stiffen as they enter mitosis. We investigated the effect of oncogene activation on this process in normal epithelial cells. We found that short-term induction of oncogenic Ras^V12^ activates downstream mitogen-activated protein kinase (MEK-ERK) signaling to alter cell mechanics and enhance mitotic rounding, so that Ras^V12^-expressing cells are softer in interphase but stiffen more upon entry into mitosis. These Ras^V12^-dependent changes allow cells to round up and divide faithfully when confined underneath a stiff hydrogel, conditions in which normal cells and cells with reduced levels of Ras-ERK signaling suffer multiple spindle assembly and chromosome segregation errors. Thus, by promoting cell rounding and stiffening in mitosis, oncogenic Ras^V12^ enables cells to proliferate under conditions of mechanical confinement like those experienced by cells in crowded tumors.

## Highlights

• Ras-ERK signaling controls cell shape in mitosis
• Activation of Ras alters cell mechanics to enhance mitotic stiffening
• Cells expressing Ras are better able to round up to divide under stiff gels
• Ras limits DNA segregation errors normally observed in confined cell division

## Graphical Abstract

**Figure:**
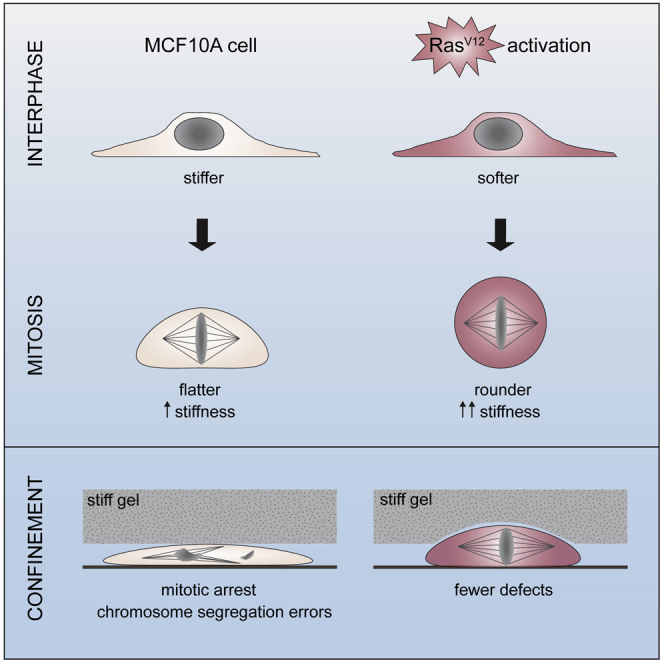

## Teaser

Matthews et al. show that activation of the oncogenic Ras-ERK signaling pathway changes the shape and mechanical properties of normal epithelial cells in mitosis. This enables Ras-activated cells to round up and accurately segregate chromosomes when dividing in conditions of mechanical confinement.

## Introduction

Animal cells undergo profound changes in cell shape and mechanics at the start of mitosis. In tissue culture, adherent spread cells retract their margins in early mitosis and round up to become spherical (Ramkumar and Baum, 2016)—a process driven by a combination of substrate de-attachment (Dix et al., 2018), actomyosin remodeling (Kunda et al., 2008, Maddox and Burridge, 2003, Matthews et al., 2012), and osmotic swelling (Son et al., 2015, Stewart et al., 2011, Zlotek-Zlotkiewicz et al., 2015). At the same time, cells become stiffer (Fischer-Friedrich et al., 2016, Kunda et al., 2008, Matthews et al., 2012). This change in cell mechanics requires the remodeling of actin filaments into a thin network at the cell cortex (Chugh et al., 2017) and is essential for cells to divide in a stiff gel that mimics a tissue environment (Nam and Chaudhuri, 2018). Limiting mitotic rounding by physical confinement results in defects in spindle formation and chromosome segregation (Lancaster et al., 2013) as flattened cells lack the 3-dimensional (3D) space required to assemble a bipolar spindle and capture chromosomes (Cadart et al., 2014).

While almost all proliferating animal cells undergo a degree of mitotic rounding, different cell types exhibit striking differences in the extent to which they round (Cadart et al., 2014, Ramkumar and Baum, 2016). In this context, we previously noted that cancer cell lines tend to round up more than many non-transformed cells (Dix et al., 2018). There are two likely explanations for this. First, the ability of a cell to successfully build a spindle in a flattened state depends on centrosome number and DNA content (Cadart et al., 2014, Lancaster et al., 2013). This is important since cancer cells tend to have more chromosomes and centrosomes than non-transformed cells. HeLa cells, for example, have close to three times the normal number of chromosomes (Adey et al., 2013). In line with this, cancer cells suffer greater mitotic defects than non-transformed cells when rounding is limited by mechanical constraints (Cadart et al., 2014, Lancaster et al., 2013). Second, while normal cells divide in a defined tissue niche where the mechanical and physical environment is tightly regulated, cancer cells must be able to divide in a wide range of environments including a crowded primary tumor, in the circulatory system (Adams et al., 2016), and at metastatic sites, all of which have biochemical and mechanical properties that are very different to those in the original tissue. While the nature of the genetic changes that enable cancer cells to divide in different environments is not known, we have previously shown that the actomyosin cytoskeleton controls mitotic rounding (Kunda et al., 2008, Lancaster et al., 2013, Matthews et al., 2012, Rosa et al., 2015). This led us to put forward the hypothesis that regulators of the actomyosin cortex may be co-opted by cancer cells to enable them to successfully divide in different environments (Matthews and Baum, 2012). Indeed, many of the proteins required for mitotic rounding, such as Ect2 and Ezrin, are upregulated in cancer (Bruce et al., 2007, Fields and Justilien, 2010). However, it is difficult to directly compare mitotic rounding and cell division in normal and cancer cells, not least because of the large number of changes that cells accumulate during transformation and cancer evolution. Therefore, as an experimental system in which to study how transformation influences mitotic rounding, we chose to induce the expression of single oncogenes in a non-transformed diploid epithelial cell line: MCF10A cells. Remarkably, in this model system, 5 h of expression of a single oncogene, Ras^V12^, was sufficient to profoundly alter mitotic cell shape dynamics and mechanics—mirroring the division changes seen in cells with sustained, long-term Ras^V12^ overexpression. At the same time, Ras^V12^ activation was able to induce mechanical changes that improved the ability of cells to round up and faithfully divide underneath gels designed to mimic a stiff extracellular environment. These data suggest that oncogenes can directly promote high-fidelity division of cells in stiff and confined environments.

## Results

### Activation of Oncogenic Ras Alters Mitotic Cell Geometry

**Figure 1:**
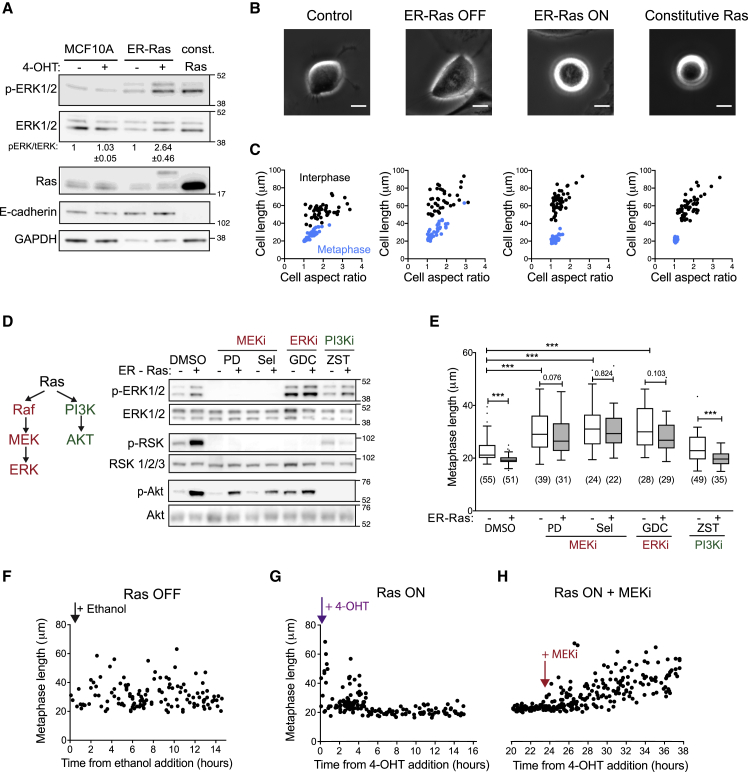
Ras/MEK/ERK Signaling Controls Cell Shape in Mitosis (A) Western blot showing levels of ERK1/2 phosphorylation and total ERK1/2, Ras, and E-cadherin expression in MCF10A, MCF10A ER-hRas^V12^, and MCF10A+hRas^v12^ cells. MCF10A and ER-hRas^V12^ cells were treated with ethanol (−) or 4-OH-tamoxifen (+) for 7 h before lysis. The ratio of pERK/tERK signal was quantified for 3 independent blots and means and standard deviation, normalized to the ethanol (−) condition, are shown. Position of molecular weight markers (kDa) is indicated on right-hand side. (B) Representative phase contrast images of the different cell types in metaphase. Metaphase is taken as 5 min before anaphase elongation or furrowing is first observed. Scale bars represent 10 μm. (C) Scatter plots of cell length (Feret diameter) and aspect ratio for the different cell types in interphase (black, taken as 15 min before nuclear envelope breakdown) and metaphase (blue, 5 min before anaphase). Cells were imaged every 5 min for 15 h using phase contrast microscopy, and the shapes were recorded for every cell division. For ER-hRas^V12^ cells, cells were analyzed 5–15 h post-ethanol or 4-OH-tamoxifen addition. n = 30 cells per condition. (D) Western blot showing levels of phospho-ERK1/2, total ERK1/2, phospho-p90RSK, total RSK1/2/3, phospho-Akt, and total Akt for MCF10A ER-hRas^V12^ cells, treated for 7 h with ethanol (−) or 4-OH-tamoxifen (+) plus DMSO or the following inhibitors: 2 μM PD 184352, 10 μM selumetinib (MEK inhibitors – MEKi), 10 μM GDC-0994 (ERK inhibitor – ERKi), and 2 μM ZSTK474 (PI3K inhibitor – PI3Ki). The position of the molecular weight markers (kDa) is indicated on the right-hand side. (E) Boxplot showing metaphase length for ER-hRas^V12^ cells following 5–15 h ethanol or 4-OH-tamoxifen treatment alongside addition of DMSO or the following small molecule inhibitors: 2 μM PD 184352, 10 μM selumetinib (MEK inhibitors), 10 μM GDC-0994 (ERKi), or 2 μM ZSTK474 (PI3Ki). p Values calculated using Mann-Whitney ^∗∗∗^p < 0.001. (F–H) Plot of the metaphase length of individual ER-hRas^V12^ cells dividing against time after ethanol (F) or 4-OH-tamoxifen (G) addition and following addition of 10 μM selumetinib (H).

To investigate the impact of oncogenic signaling on mitotic cell shape and mechanics, we used the non-transformed human epithelial cell line (MCF10A) as a model system (Soule et al., 1990). Oncogenic h-Ras^G12V^ (hereafter called Ras^V12^) was activated in these cells by constitutive overexpression or using an inducible system in which estrogen receptor (ER)-fused Ras^V12^ can be rapidly activated and stabilized following addition of 4-OH-tamoxifen (4-OHT) (Molina-Arcas et al., 2013). Overexpression of full-length or ER-fused Ras and phosphorylation of its downstream target, extracellular signal-regulated protein kinases 1/2 (ERK1/2 – the human MAPK), were confirmed for both lines using western blotting (Figure 1A). Activation of inducible, ER-fused Ras increased ERK phosphorylation over a time period of 1–10 h following 4-OHT addition (Figure S1A). To determine how cells change shape upon entry into mitosis, brightfield time-lapse microscopy was used in each case to follow unlabeled asynchronous populations of cells as they divided (Figure 1B). We quantified cell length (Feret diameter) and aspect ratio 15 min before mitotic entry and in metaphase (5 min before anaphase elongation) for each population (Figure 1C). We found that induction of Ras^V12^ significantly altered cell length, area, and aspect ratio in metaphase but not interphase cells (Figures 1C, S1B, and S1C). This effect was also seen in a cell line constitutively expressing Ras^V12^, as a model of long-term Ras activation (Figures 1C and S1C). Since activation of Ras^V12^ likely affects the stability of cell–cell junctions, we asked how this effect is influenced by the presence of neighboring cells. We observed that MCF10A cells rounded more upon entry into mitosis if cultured at a low density, compared to cells surrounded by neighbors in the middle of a monolayer. However, short-term Ras^V12^ induction enhanced mitotic rounding in single cells and cells within a monolayer (Figure S1D), which then rounded to a similar extent. This suggests that Ras activation, for as little as 5 h, alters cell/cell junctions and monolayer integrity, even though these cells continue to express E-cadherin (Figure 1A). Importantly, the difference in the rate of rounding observed in single cells suggests that Ras^V12^ also induces intrinsic changes to mitotic shape. Since the aim of this work was to investigate these intrinsic differences, all further experiments were carried out with cells plated at a low density to exclude effects of cell/cell interactions on cell shape.

### Ras Signals through the MEK/ERK Pathway to Control Mitotic Cell Shape

Two of the main effectors of Ras activation are the MAPK and phosphatidylinositol 3-kinase (PI3K) signaling cascades, and we observed increased phosphorylation of ERK, ERK-target p90-ribosomal S6 kinase (RSK), and PI3K-target Akt following Ras^V12^ activation (Figure 1D). To test whether these pathways control mitotic shape, we inhibited different elements of the pathways in cells with and without Ras^V12^ induction. Inhibition of mitogen-activated protein kinase kinase (MEK) or ERK, but not PI3K, abolished the difference in mitotic cell shape following Ras activation (Figure 1E). MEK or ERK inhibition also significantly increased metaphase cell length in uninduced ER-hRas^V12^ and control MCF10A cells (Figures 1E and S2A), likely by inhibiting the low, basal levels of ERK activity observed in these proliferating cells (Figure 1A). Conversely, MEK and ERK inhibitors altered mitotic shape in constitutive Ras^V12^-expressing cells (Figure S2B), which have a high basal level of ERK phosphorylation (Figure 1A). This reveals a previously unidentified role for the Ras-ERK signaling cascade in controlling mitotic rounding. We examined the timing of these Ras-ERK-dependent changes in mitotic cell shape and found that the change in metaphase length began around 5 h after Ras^V12^ activation (Figures 1F and 1G) and was reversed within 30 min of treatment with a MEK inhibitor (Figure 1H). These data demonstrate that the Ras-ERK signaling pathway plays a role in controlling the shape of cells in mitosis. More surprisingly, these changes emerge over a timescale of minutes to hours and are not simply a long-term consequence of oncogenic transformation.

### Ras-ERK Signaling Controls Mitotic Cell Shape in Cancer Cell Lines

Having uncovered a role for Ras-ERK signaling in determining mitotic shape in normal epithelial cells, we next asked whether it plays a similar role in Ras-transformed cancer cell lines. The MCF10A-CA1 cell line was derived from MCF10A cells by transformation with h-Ras followed by selection for aggressive tumor formation in mice xenografts (Santner et al., 2001). We imaged these cells using time-lapse microscopy and measured cell length in mitosis. As with their non-transformed counterparts, we found that inhibitors of MEK or ERK significantly increased metaphase length (Figure S2C). Since k-Ras is the Ras isoform most commonly mutated in human cancers, we asked whether it was able to promote rounding in a similar way to h-Ras. We confirmed that inducible activation of k-Ras^V12^ in MCF10A cells (Molina-Arcas et al., 2013) also enhanced rounding (Figure S2D). Similarly, inhibition of MEK or ERK decreased metaphase length in Capan-1 cells (Figures S2E–S2G), a transformed cell line originating from a human metastatic pancreatic ductal adenocarcinoma, which harbors two k-Ras^G12V^ mutant alleles. These data suggest that the Ras-ERK signaling pathway functions to promote mitotic rounding in both non-transformed cells and Ras-mutated cancer cells. Overall, we saw a correlation between pathway activity levels (as measured by ERK phosphorylation) and roundness in mitosis, with cells with higher levels (Ras-activated MCF10A and Ras-transformed cancer cells) rounding up more at mitosis and cells with lower levels (non-transformed MCF10A and all cell types when treated with MEK or ERK inhibitors) remaining more spread.

### The Effect of Ras-ERK Signaling on Mitotic Shape Requires Actomyosin Contractility

**Figure 2:**
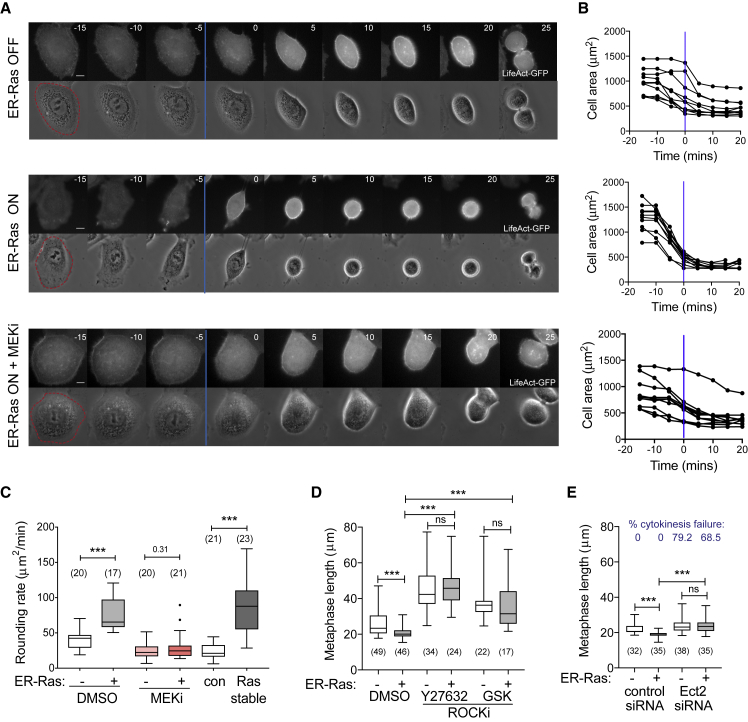
Ras/MEK/ERK Signaling Alters Contractility in Early Mitosis (A) Representative montage time-lapse images of MCF10A ER-hRas^V12^ cells labeled with LifeAct-GFP following an 8 h treatment with ethanol, 4-OH-tamoxifen, or 4-OH-tamoxifen + 2 μM PD 184352 (MEKi). Time in minutes is aligned so that t = 0 is the frame after NEB (blue line). Scale bars represent 10 μm. (B) Quantification of cell area for 8 cells from conditions in (A) entering mitosis, aligned so that t = 0 is NEB (blue lines). Measurements were taken from time-lapse microscopy of cells 5–15 h after treatment. (C) Boxplot showing rate of area decrease during mitotic rounding for MCF10A ER-hRas^V12^ cells following 8 h treatment with ethanol or 4-OH-tamoxifen and DMSO or 2 μM PD 184352 as well as control, unsynchronized MCF10A, and MCF10A+hRas^v12^ cells. p Values calculated using Mann-Whitney ^∗∗∗^p < 0.001. (D and E) Boxplots showing metaphase cell length for ER-hRas^V12^ cells following 5–15 h ethanol or 4-OH-tamoxifen treatment and treated with DMSO, 25 μM Y-27632, or 2 μM GSK 269962 (ROCK inhibitors) (D) or scrambled or Ect2 siRNA (E). Ect2 knockdown was verified by analyzing the cytokinesis failure rate (shown in blue). p Values calculated using Mann-Whitney ^∗∗∗^p < 0.001.

To determine how the dynamics of mitotic rounding was altered following Ras activation, we constructed MCF10A lines that stably express LifeAct-GFP to visualize actomyosin dynamics through mitosis (Figure 2). By following the shape changes of individual cells, we observed that mitotic rounding begins 5–10 min before nuclear envelope breakdown (NEB) (Figures 2A and 2B; Videos S1, S2, and S3) as previously observed in HeLa cells (Matthews et al., 2012). Rounding was initiated at a similar time in cells with or without Ras^V12^ induction, but Ras^V12^-expressing cells rounded at a faster rate, especially before NEB (Figure 2B). This was the case for cells induced to express Ras^V12^ and for cells overexpressing constitutive Ras^V12^ (Figure 2C). Thus, cells expressing oncogenic Ras^V12^ were almost completely round by pro-metaphase while control cells or cells treated with a MEK inhibitor continued to round up throughout mitosis until they entered anaphase and divided. This was reflected in a significant difference in rounding rates, with Ras cells rounding faster than controls; a difference that was abolished by treatment with a MEK inhibitor (Figure 2C).

Video S1. Control Cell Division, Related to Figure 2A Time-lapse phase contrast (left) and fluorescent (right) movie of a MCF10A ER-hRas^V12^ cell labeled with LifeAct-GFP undergoing mitotis after treatment with ethanol (uninduced). Time is in minutes. Scale bar is 10 μm.

Video S2. Ras^V12^-Activated Cell Division, Related to Figure 2A Time-lapse phase contrast (left) and fluorescent (right) movie of a MCF10A ER-hRas^V12^ cell labeled with LifeAct-GFP undergoing mitotis after treatment with 4-OH-tamoxifen for 8 h (Ras-ON). Time is in minutes. Scale bar is 10 μm.

Video S3. MEK Inhibitor-Treated Cell Division, Related to Figure 2A Time-lapse phase contrast (left) and fluorescent (right) movie of a MCF10A ER-hRas^V12^ cell labeled with LifeAct-GFP undergoing mitotis after treatment with 4-OH-tamoxifen + 2 μM PD 184352 (MEK inhibitor) for 8 h. Time is in minutes. Scale bar is 10 μm.

Since Ras activation did not affect cell volume in mitosis (Figure S3A), mitotic swelling (Son et al., 2015, Stewart et al., 2011, Zlotek-Zlotkiewicz et al., 2015) appeared unaltered. Instead, it is likely that the Ras-ERK pathway changes actomyosin contractility. To test this, we treated cells with an inhibitor of Rho kinase (ROCK). ROCK inhibition abolished the effect of Ras on cell shape as well as dramatically increasing mitotic length in all conditions (Figure 2D). This fits with the previously well-described requirement for ROCK and actomyosin contractility in mitotic rounding (Maddox and Burridge, 2003, Matthews et al., 2012). Since ROCK inhibition is a global treatment that will disrupt contractility in all stages of the cell cycle, we asked whether actomyosin contractility was required specifically at mitotic entry. Actin re-arrangements in early mitosis are driven by the activation and re-localization of RhoGEF, Ect2 (Matthews et al., 2012), which activates RhoA (Maddox and Burridge, 2003), and ROCK to contract the cell edge. Again, inhibition of Ect2 using siRNA knockdown removed any difference between uninduced and Ras-induced cells (Figure 2E). Taken together, these data show that activation of the oncogenic Ras-ERK signaling pathway is sufficient to induce changes in mitotic cell shape in epithelial cells over both short and long timescales, in a manner that depends on actomyosin contractility.

### Ras Activation Alters Cell Mechanics

**Figure 3:**
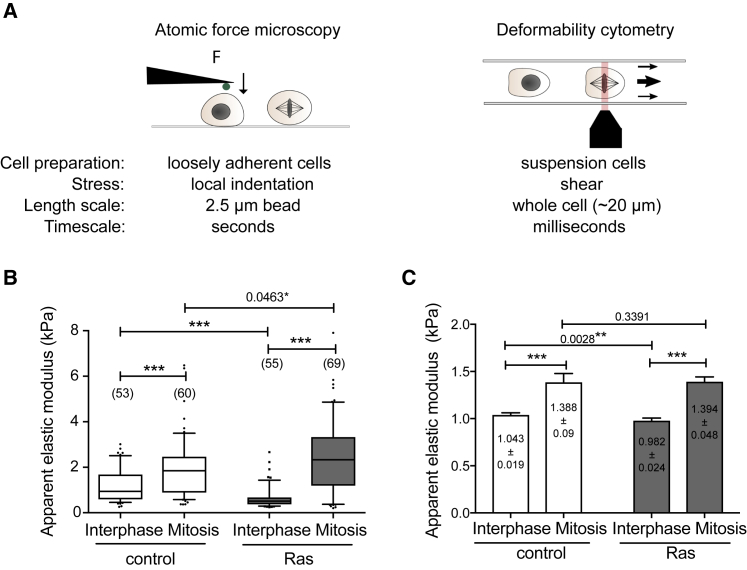
Ras Activation Alters Cell Mechanical Properties (A) Schematic comparing two different techniques used to measure cell mechanical properties: atomic force microscopy (AFM) and real-time deformability cytometry (RT-DC). (B) Boxplot showing the apparent elastic modulus for rounded control MCF10A cells and MCF10A+hRas^v12^ in interphase and mitosis (arrested in pro-metaphase by S-trityl-L-cysteine (STLC) treatment) as determined by AFM. The number of curves analyzed are indicated in brackets. Data shown is from one representative experiment (n = 2). (C) Bar chart shows apparent elastic modulus (mean + SEM) calculated using RT-DC for interphase and mitotic cells from 10 independent experiments (n > 15,000 cells). Because of the large sample size, a linear mixed model was used to calculate the statistical significance of the results. ^∗∗∗^p < 0.001.

The remodeling of the actomyosin cortex at mitotic entry alters both cell shape and cell mechanics. As a result, mitotic cells are significantly stiffer than interphase cells (Fischer-Friedrich et al., 2016, Kunda et al., 2008, Matthews et al., 2012). This led us to compare the mechanical properties of normal MCF10A cells and Ras^V12^-overexpressing cells using two different but complementary techniques (Figure 3A). First, we measured the apparent elastic modulus of individual cells using atomic force microscopy (AFM). To control for the effect of cell shape on mechanics, cells were first removed from the substrate using a brief treatment with trypsin-EDTA and then allowed to re-attach but not spread. In this way, we were able to compare the stiffness of interphase and mitotic cells with the same shape (Figure 3A). Under these conditions, interphase Ras^V12^-expressing cells were significantly softer than interphase controls, as previously reported for rounded cells (Gullekson et al., 2017). At the same time, Ras^V12^-expressing cells were marginally but significantly stiffer than control cells in mitosis (Figure 3B). As a consequence, Ras^V12^ cells underwent a greater fold increase in their elastic modulus upon mitotic entry than controls (4.21× mitotic increase for Ras^V12^ cells and 2.23× mitotic increase for controls). We then used real-time deformability cytometry (RT-DC) (Rosendahl et al., 2018) as a complementary approach to measure cell stiffness (Figure 3C). Whereas AFM probes the mechanics of a small region at the cell cortex of an adherent cell, RT-DC is a high-throughput method (>2,000 cells per experiment) to infer whole-cell mechanical properties of suspended cells based on their deformation under shear flow. Because of the differences in the length and timescales of measurement (Figure 3A), it is not possible to compare absolute measurements of stiffness obtained using these two techniques but one would expect to observe similar trends using both methods. Using RT-DC, we confirmed that Ras^V12^-expressing cells were significantly softer than their non-transformed counterparts in interphase but were of similar stiffness in mitosis (Figure 3C). These data show that long-term Ras overexpression results in a greater increase in stiffness when cells enter mitosis, as they start from a softer interphase state but ultimately become at least as stiff as controls in metaphase and that this occurs in a manner that is independent of their shape.

### Ras Activation Promotes Mitotic Rounding under Confinement

**Figure 4:**
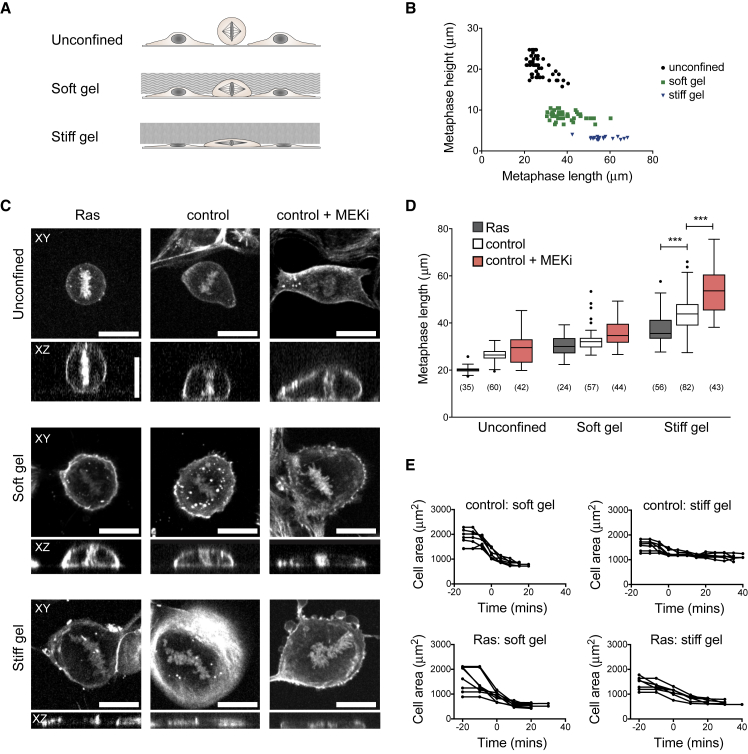
Ras Activation Promotes Mitotic Rounding under Confinement (A) Schematic of cells dividing in confinement under compliant (~5 kPa) and stiff (~ 30 kPa) polyacrylamide gels. (B) Plot of maximum metaphase length versus height for unconfined MCF10A cells and cells under soft (~5 kPa) and stiff (~ 30 kPa) gels. Measurements were taken from confocal time-lapse images as shown in (C). Metaphase was taken as the frame before anaphase. (C) XY and XZ confocal slices of metaphase MCF10A+hRas^v12^ cells, MCF10A cells, and MCF10A cells treated for 5–10 h with 10 μM selumetinib. Cells were pre-treated with 100 nM SiR-DNA and SiR-actin to label DNA and actin. Scale bars represent 20 μm. (D) Boxplot showing metaphase cell length for MCF10A+hRas^v12^, control MCF10A, and MCF10A + 2 μM PD 184352 without confinement and under soft and stiff gels. Data come from time-lapse movies for 15 h after confinement onset. The MEKi was added immediately before confinement and mitoses were analyzed 5–15 h post addition and confinement. p Values calculated using Mann-Whitney ^∗∗∗^p < 0.001. (E) Graphs showing cell area for control MCF10A cells and MCF10A+hRas^v12^ entering mitosis under soft and stiff gels, aligned so that t = 0 is NEB.

Next, we sought to investigate how these changes in the rate and extent of rounding and cell mechanics induced by Ras^V12^ influence cell division under confinement. To do this, we confined cells under polyacrylamide hydrogels of different stiffness (Le Berre et al., 2014) a compliant gel of around ∼5 kPa and a stiff gel of around ∼30 kPa (Figure 4A) and filmed cells as they proceeded through mitosis in these confined conditions. Under the soft gel, cells were able to round up partially at mitosis, with a decrease in mean metaphase cell height from 20 to 8.5 μm in wild-type MCF10A cells (Figures 4B and S3B). By contrast, under the stiffer gel, mitotic rounding was severely limited with cells unable to increase their height beyond 3 μm (Figures 4B and S3B). Strikingly, however, the expression of Ras^V12^ improved the ability of cells to round up under both soft and stiff gels (Figures 4C and 4D). This led to a significant reduction in metaphase length under a stiff gel compared to controls (Figure 4D, mean length was 44.2 ± 7.6 μm for controls and 37.5 ± 6.2 μm for Ras^V12^ cells). Conversely, cells treated with a MEK inhibitor remained even flatter when confined under the stiff gel (Figures 4C and 4D, mean length 55.1 ± 12.5). This was reflected in a change in the rate of cell area decrease, with Ras^V12^-expressing cells able to retract their margins faster (Figure 4E). As these experiments were carried out using cells constitutively overexpressing Ras^V12^, we asked whether short-term Ras activation was able to promote rounding under confinement. We found that activation of Ras^V12^ for 5–15 h was sufficient to reproduce the differences observed using the constitutive line by significantly reducing metaphase length under both soft and stiff gels, an effect that was blocked by addition of the MEK inhibitor (Figure S3C). Thus, the alterations in cell shape and contractility induced by both short- and long-term Ras^V12^ activation allow cells to round up better in confined conditions, suggesting that they are able to exert more force to deform the overlying gel as they enter mitosis.

### Ras Enhanced Mitotic Rounding Reduces Mitotic Defects under Confinement

**Figure 5:**
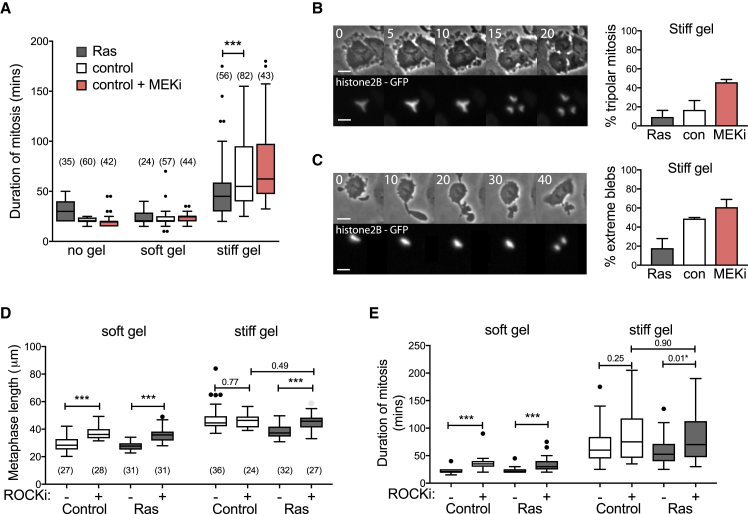
Ras Activation Reduces Mitotic Defects in Confinement (A) Boxplot showing the time taken for cells to progress through mitosis (from NEB to anaphase) for MCF10A+hRas^v12^, control MCF10A, and MCF10A + 2 μM PD 184352 without confinement and under soft and stiff gels. Data come from time-lapse movies for 15 h after confinement onset. MEKi was added immediately before confinement and mitoses were analyzed 5–15 h post-addition/confinement. p Values calculated using Mann-Whitney ^∗∗∗^p < 0.001. (B) Time-lapse montage showing an example of tri-polar mitosis and graph showing the percentage of cells (mean ± SD) that undergo tri-polar mitosis underneath a stiff gel. n = 3 independent experiments. (C) Time-lapse montage showing an example of extreme blebbing and graph showing the percentage (mean ± SD) of cells that undergo extreme blebbing underneath a stiff gel. n = 3 independent experiments. Extreme blebbing was defined as any mitotic cells having a bleb equal to or longer than cell diameter. Note that no tri-polar mitosis or extreme blebbing was observed in any condition under soft gels or without gels. Times are in minutes and scale bars represent 20 μm. (D and E) Boxplot of metaphase length (D) and mitotic duration (time from NEB to anaphase) (E) for control MCF10A and MCF10A+hRas^v12^ cells under soft and stiff gels, treated with DMSO or 25 μM Y-27632 (ROCK inhibitor – ROCKi). n for (E) is the same as indicated in the figure in (D).

Limiting mitotic rounding by confinement results in multiple mitotic defects (Lancaster et al., 2013). This led us to test whether the enhanced ability of Ras^V12^ cells to round up under a stiff gel aided cell division under these conditions. To do so, we first measured the time taken by cells to pass through mitosis (NEB to anaphase) under gels (Figure 5A). This serves as a good proxy for spindle defects, as anaphase is only able to proceed once all chromosomes are attached to the spindle to satisfy the spindle assembly checkpoint (Musacchio, 2015). When cells were confined under a soft gel, we did not observe any change in the timing of mitosis. However, increasing gel stiffness resulted in a prolonged mitosis in all conditions (Figure 5A). Ras^V12^ cells, which were better able to round up under the gel (Figures 4C–4E), were significantly faster at progressing through mitosis compared to normal MCF10A cells (Figure 5A). These observed delays in mitotic progression were associated with profound differences in spindle integrity under stiff gels. Under the stiff gel, spindles often fractured to produce a tri-polar spindle (Lancaster et al., 2013), resulting in a cell dividing into three rather than two daughter cells. The incidence of this type of spindle fracture decreased in Ras^V12^-expressing cells and increased following treatment with a MEK inhibitor (Figure 5B). We also observed signs of cortical instability such as extreme blebs in mitotic cells under stiff gels. These blebs were often longer than the cell itself, and were sometimes seen breaking off from the cell body. Again, this type of extreme blebbing was reduced in cells expressing Ras^V12^ compared to normal MCF10A and cells in which MEK had been inhibited (Figure 5C). These data suggest that Ras^V12^ expression is able to limit some of the mitotic defects induced by confinement. To test if this is due to the Ras-dependent changes in mitotic rounding, we repeated the analysis in cells in which actomyosin contractility was inhibited using a ROCK inhibitor. Under these conditions, mitotic rounding was limited under both soft and stiff gels (Figure 5D), causing cells to undergo a prolonged mitosis (Figure 5E) relative to times observed for the non-confined control (Figure S3D). Crucially, the ROCK inhibitor also abolished any difference between Ras^V12^-expressing and control cells (Figure 5D). This demonstrates that the actomyosin cytoskeleton is essential to generate the pushing force that enables Ras^V12^ cells to round up and successfully divide in confined conditions.

## Discussion

Oncogenic Ras is a driver mutation in many cancer types that leads to multiple downstream events to promote transformation (Simanshu et al., 2017). Here, we have discovered a role for Ras in directly controlling cell geometry, mechanics, and force generation during mitosis. By using non-transformed cells in which Ras^V12^ can be inducibly activated as a model system, we have shown that the activation of Ras^V12^ for a short time period (5 h) is sufficient to change cell shape in mitosis. Since the effects of Ras expression could be reversed by inhibition of the MEK/ERK pathway, this reveals a direct correlation between Ras-ERK pathway signaling and mitotic cell rounding. Moreover, this was also the case for non-transformed cells and a k-Ras-mutated pancreatic cancer cell line, implying that oncogenic Ras simply hijacks and amplifies the normal impact of Ras-ERK signaling on cell division. As there are likely additional mechanisms at play in cancer cells due to the large number of mutations accumulated during cancer evolution, it will be important in the future to investigate the extent to which Ras mutations account for changes in cell division in the context of a developing tumor in animal models or human tissue. As a simple model in which to explore the impact of oncogenic Ras on the ability of cells to divide in different mechanical environments, we challenged cells to divide when confined under soft and stiff hydrogels. We found that Ras activation enables mitotic cells to round up underneath hydrogels that are sufficiently stiff to limit the rounding of control cells. Strikingly, in this way, Ras^V12^ reduces the incidence of mitotic defects, allowing Ras-expressing cells to complete a more timely and accurate segregation of chromosomes in confined conditions.

In these cells, as in other systems, activation of the Ras-ERK signaling cascade likely leads to a suite of different cellular changes including the direct phosphorylation of ERK target proteins (Carlson et al., 2011), changes to transcription (Wilson et al., 2017), substrate adhesion (Pawlak and Helfman, 2002, Schäfer et al., 2016), and the actin cytoskeleton (Mendoza et al., 2015, Pawlak and Helfman, 2002). While we have not identified the precise molecular mechanisms by which Ras alters mitotic shape, it is clear from our data that these changes depend on actomyosin contractility. This fits with the well-documented role of the Ras-ERK pathway in controlling interphase actin filament organization and contractility to promote cell motility and invasion (Choi and Helfman, 2014, Mendoza et al., 2015, Pollock et al., 2005). High levels of k-Ras expression in lung cancer cells induces myosin-dependent cell shape changes in interphase with cells adopting a rounded, sphere-like morphology (Schäfer et al., 2016). Recent work has shown that activation of Ras in pancreatic epithelial cells leads to changes in the apico-basal distribution of myosin and interphase cell shape in a way that contributes to cancer morphogenesis (Messal et al., 2019). Ras-ERK signaling has been shown to directly modulate the activity of RhoA (Chen et al., 2003, Makrodouli et al., 2011, Sahai et al., 2001, Tong et al., 2016) and myosin phosphorylation (Nguyen et al., 1999, Samson et al., 2019). However, given the 5 h delay between Ras activation and visible changes in mitotic rounding, we think it likely that many of the effects of Ras in this case are mediated by a complex set of effectors, as appears to be the case in pancreatic epithelial cells (Messal et al., 2019). This conclusion is supported by the fact that the effects of Ras activation are manifold and include interphase softening, an increase in the rate of mitotic rounding, and changes to how cells re-spread after division (Mali et al., 2010). Ras has been shown to affect interphase cell mechanics; inducible activation softens or stiffens cells in a substrate-dependent manner (Gullekson et al., 2017, Linthicum et al., 2018) while long-term overexpression softens interphase cells (Lin et al., 2015), as we have observed here. Furthermore, ERK signaling activated by induction of oncogenic Src can also change cell mechanics by changing the expression levels of multiple actin-binding proteins after several hours (Tavares et al., 2017). Here, we have found that Ras overexpression softens cells at interphase, resulting in a greater increase in stiffness when cells enter mitosis. This likely allows them to exert more force outward as they enter mitosis, as has also been observed in epithelial cells forced to undergo an epithelial-to-mesenchymal transition (Hosseini et al., 2019).

The function of these oncogene-induced changes to mitotic geometry and mechanics become most apparent when cells are challenged to divide underneath a stiff gel. This illustrates the importance of considering environmental context when studying cell division. In a 2D tissue culture, mitotic rounding primarily requires loss of substrate adhesion. However, under confinement, the cell’s ability to generate force to push against its environment is crucial (Lancaster et al., 2013). This is also the case in a 3D culture, where mitotic rounding generates force in all directions (Nam and Chaudhuri, 2018), similar to cells in the tissue context. Environmental context is also important for the fidelity of cell division: recent work has shown that primary epithelial cells cultured in 3D suffer fewer chromosome segregation defects than in 2D (Knouse et al., 2018). Conversely, a stiff 3D gel that limits anaphase elongation also induces chromosomal segregation defects (Nam and Chaudhuri, 2018). This is especially relevant to tumors where profound changes to tissue integrity and mechanical properties (Nagelkerke et al., 2015, Plodinec et al., 2012) have been proposed to impact the cell division process (Matthews and Baum, 2012). Here, we have shown that oncogenic Ras-ERK signaling enables cells to overcome mechanical constraints in their local environment that would otherwise impair their ability to divide. In the future, it will be important to explore whether Ras promotes faithful and accurate cell division in a similar way in Ras-transformed cancer cells and whether this is part of a more general mechanism by which oncogenic signaling can facilitate cell division in confined or stiff environments, such as those found in tumors.

## STAR★ Methods

### Key Resources Table

**Table:** 

REAGENT or RESOURCE	SOURCE	IDENTIFIER
**Antibodies**		

Rabbit polyclonal anti-phospho-ERK1/2 (Thr202/Tyr204)	Cell Signaling Technology	Cat# 9101 RRID: AB_331646
Rabbit polyclonal anti-p44/42 MAPK (ERK1/2)	Cell Signaling Technology	Cat# 9102, RRID: AB_330744
Rabbit polyclonal anti-p90RSK, phospho (Ser380)	Cell Signaling Technology	Cat# 9341, RRID: AB_330753
Rabbit monoclonal Anti-RSK1 / RSK2 / RSK3, Clone 32D7	Cell Signaling Technology	Cat# 9355, RRID: AB_659900
Mouse monoclonal Anti-Akt1, Clone 5C10	Santa Cruz Biotechnology	Cat# sc-81434, RRID: AB_1118808
Rabbit polyclonal anti-Ras	Cell Signaling Technology	Cat# 3965, RRID: AB_2180216
Rat monoclonal anti-E-cadherin (ECCD2)	Thermo Fisher Scientific	Cat# 13-1900, RRID: AB_2533005
Mouse monoclonal anti-GAPDH (GA1R)	Thermo Fisher Scientific	Cat# MA5-15738, RRID: AB_10977387

**Bacterial and Virus Strains**		

rLV-Ubi-LifeAct-GFP2 lentivirus	Ibidi	Cat# 60141

**Chemicals, Peptides, and Recombinant Proteins**		

PD 184352	Cell Signaling Technology	Cat# 12147
Selumetinib	Selleckchem	Cat# S1008
GDC-0994	Selleckchem	Cat# S755403
ZSTK474	Selleckchem	Cat# S1072
Y-27632	Sigma-Aldrich	Cat# Y0503
GSK 269962	Tocris	Cat# 4009 CAS: 850664-21-0

**Experimental Models: Cell Lines**		

MCF10A	Horizon	RRID: CVCL_0598
MCF10A+ constitutive hRas^G12V^	S. Godinho	N/A
MCF10A-CA1	E. Sahai (Santner et al., 2001)	N/A
MCF10A-ER:hRas^G12V^	J. Downward (Molina-Arcas et al., 2013)	N/A
MCF10A-ER:kRas^G12V^	J. Downward (Molina-Arcas et al., 2013)	N/A
MCF10A-ER:hRas^G12V+^ LiveAct-GFP	This paper	N/A
Capan-1	Cell Services at the Francis Crick Institute	RRID: CVCL_0237

**Oligonucleotides**		

Hs_ECT2_6 Flexitube siRNA ATGACGCATATTAATGAGGAT	Qiagen	SI03049249
AllStars negative control siRNA	Qiagen	1027280

**Software and Algorithms**		

Fiji	(Schindelin et al., 2012)	https://fiji.sc/
Prism 7	GraphPad	https://www.graphpad.com/
ShapeOut	Zell Mechanik Dresden	https://github.com/ZELLMECHANIK-DRESDEN/ShapeOut
R	RCore Team	www.R-project.org/

### Lead Contact and Materials Availability

Further information and requests for resources and reagents should be directed to and will be fulfilled by the Lead Contact, Helen Matthews (h.matthews@ucl.ac.uk). Cell lines are available upon request from the authors.

### Experimental Model and Subject Details

#### Cell Lines and Culture

MCF10A cell lines (female) were cultured in DMEM F-12 Glutamax, with 5% horse serum (Invitrogen), 20ng/ml EGF (Peprotech), 0.5mg/ml Hydrocortisone (Sigma), 100ng/ml Cholera toxin (Sigma), 10μg/ml Insulin (Sigma), 1% Penstrep (Gibco) at 37°C with 5% CO_2_. For ER-inducible cell lines, DMEM F-12 without phenol and charcoal-stripped horse serum were used. Cell lines used were MCF10A (Horizon), MCF10A+ constitutive hRas^G12V^ (gift from Susana Godinho), MCF10A-CA1 (gift from Erik Sahai (Santner et al., 2001)) and the inducible lines MCF10A-ER:hRas^G12V^ and MCF10A-ER:kRas^G12V^ (gifts from Julian Downward) (Molina-Arcas et al., 2013). Ras was activated in inducible lines by addition of 100nM 4-OH-tamoxifen (Sigma). LifeAct-GFP labeled lines were produced by infection with puromycin resistant lentivirus (rLV-Ubi-LifeAct-GFP2, Ibidi 60141). GFP positive cells were sorted using flow cytometry to produce a polyclonal stable pool. Capan-1 cells (male) were obtained from The Francis Crick Institute Cell Services facility and cultured in IMEM + 20% FBS at 37°C with 5% CO_2_. All cell lines used in this study were authenticated using STR profiling (ATCC).

### Method Details

#### Drug Treatments

The following small molecule inhibitors were used in this study: MEK inhibitors: 2 μM PD 184352 (Cell Signaling 12147) and 10 μM Selumetinib (Selleckchem S1008), ERK inhibitor: 10 μM GDC-0994 (Selleckchem S755403), PI3k inhibitor: 2 μM ZSTK474 (Selleckchem S1072), ROCK inhibitors: 25 μM Y-27632 (Sigma Y0503) and 2 μM GSK 269962 (Tocris 4009). Details of treatment times are described in figure legends.

#### siRNA Transfection

For Ect2 knockdown, Hs_ECT2_6 (ATGACGCATATTAATGAGGAT-Qiagen SI03049249) was used as previously described (Dix et al., 2018, Matthews et al., 2012) and compared to the AllStars negative control siRNA (Qiagen 1027280). Cells were transfected using Lipfectamine RNAimax (Invitrogen 13778-075) and imaged from 24 hours post transfection.

#### Live Cell Imaging

Cells were plated in fibronectin-coated, glass-bottomed plates (Mattek) 24 hours before imaging. Widefield timelapse imaging was carried out using a Nikon Ti inverted microscope or a Zeiss Axiovert 200M microscope at 5 minute timepoints using a 20x or 40x objective. Live confocal imaging was carried out using a Nikon TiE inverted stand attached to a Yokogawa CSU-X1 spinning disc scan head using a 100x objective. Cell shape descriptors were measured using FIJI (Schindelin et al., 2012, https://fiji.sc/) following manual segmentation of cell area.

#### Atomic Force Microscopy

To enrich for mitotic cells, cells were treated for 12-15 hours with 5 μM STLC (Sigma 164739). Then, the fraction containing loose mitotic cells was washed off and recombined with interphase cells that had been detached using Accutase (PAA laboratories). Cells were stained with Hoechst (Invitrogen) for 10 minutes at 37°C, washed twice and re-suspended in CO_2_-independent medium (LifeTechnologies). Cells were seeded into glass bottom dishes (FluoroDish™, WPI) and probed once they were adhered but not spread. During the AFM indentation experiment, interphase and mitotic cells were distinguished using epifluorescence images of the Hoechst stained cells. For AFM indentation measurements, a Nanowizard I equipped with a CellHesion module (JPK Instruments) was used. Arrow-T1 cantilevers (Nanoworld) were modified with polystyrene beads (radius 2.5μm, microparticles GmbH) with the aid of epoxy glue to obtain a well-defined indenter geometry and decrease local strain during indentation. Cantilevers were calibrated prior to experiments using built-in procedures of the SPM software (JPK Instruments). The bead was lowered at a defined speed (10μm/sec) onto the cell surface. After reaching the setpoint of 2nN, the cantilever was retracted. During the force-distance cycle, the force was recorded for each piezo position. The resulting force-distance curves were transformed into force- versus- tip sample separation curves (Radmacher, 2002) and fitted with the Hertz/Sneddon model for a spherical indenter (Sneddon, 1965) using the JPK data processing software (JPK DP, JPK Instruments). A Poisson ratio of 0.5 was used for the calculation of the apparent elastic modulus. Since rounded cells were probed, apparent elastic moduli were corrected according to Glaubitz et al (Glaubitz et al., 2014) using the average cell diameters of each cell population that had been determined in phase contrast images using FIJI. Each cell was probed three times, and an average apparent Young’s modulus calculated. As a control, cells that had not been treated with STLC nor Hoechst were probed and phase contrast was used to identify mitotic cells.

#### Real-time Deformability Cytometry

Mitotic cells were collected after 5 hours of STLC treatment (5μM) using the mitotic shake-off method. Interphase cells were detached by treatment with Accutase (PAA laboratories) for 15 minutes. The cells were washed with PBS and resuspended in 0.5% methylcellulose buffer before loading in the RT-DC setup as described previously (Otto et al., 2015). Cells were deformed by flowing them through 30μm × 30μm channels with a speed of 0.16μl/sec or 0.32μl/sec and the deformation was recorded using ShapeIn software (Zell Mechanik Dresden). Data for over 2000 cells was recorded for each of 10 biological replicates of the experiment. Apparent elastic modulus was calculated using ShapeOut Software (Zell Mechanik Dresden, available at https://github.com/ZELLMECHANIK-DRESDEN/ShapeOut) based on a numerical simulation model described previously (Mokbel et al., 2017). The results from 10 independent experiments were analysed using a Linear Mixed Model method integrated in ShapeOut Software (Zell Mechanik Dresden) (Herbig et al., 2018).

#### Polyacrylamide Gel Confinement

Polyacrylamide gels were polymerized on 18mm glass coverslips. Coverslips were first functionalized by plasma cleaning for 30s (Diener Femto), followed by incubation with a solution of 0.3 % Bind-Silane (Sigma M6514)/5% acetic acid in ethanol. Coverslips were then rinsed with ethanol and dried with compressed air. Polyacrylamide gel solutions were made up as 1 mL solutions in PBS as follows: Stiff gels (∼30 kPa): 250 μL acrylamide 40% (Sigma), 100 μL bisacrylamide 2% (Fisher Scientific), 10 μL APS (10% in water, Sigma), 1 μL TEMED (Sigma). Soft gels (∼5 kPa): 187.5 μL acrylamide, 30 μL bisacrylamide, 10 μL APS and 1 μL TEMED. Following TEMED addition, 200 μL of gel solution was immediately pipetted onto a flat Perspex plate and a functionalized coverslip was placed on top. Following polymerization, gels were removed from the Perspex using a square-edged scalpel and hydrated by incubation in PBS for 1-2 hours. Gels were then incubated with cell culture media overnight before use. Where drug treatments were used, these were also included in pre-incubation media. Cells were plated in plastic 12-well plates (Thermo Scientific) 24 hours before a confinement experiment. Where two different cell lines were compared (eg MCF10A and MCF10A+Ras^V12^), both were plated in the same well separated by a PDMS divider that was removed before confinement. This allowed direct comparison of different cell types under the same gel. To confine the cells, the polyacrylamide-coated coverslips were attached to soft PDMS (1:40 cross-linker: base mix) cylinders of approx. 17mm height and 15mm diameter, which were then attached to the lid of the plate, lowered onto the cells and secured with tape. This method of confinement is described in detail by Le Berre et al (Le Berre et al., 2014). To obtain confocal images and measure cell heights, gel confinement was carried out using a dynamic cell confinement method. For this, cells were seeded onto a fibronectin coated 35 mm glass bottom dish (MatTek) overnight in growth medium. To visualize DNA and actin, cells were pre-incubated for 6-12 hours with 100 nM SiR-DNA and SiR-Actin (Spirochrome) prior to imaging. For MEK inhibition, cells were pre-treated for 5-7 hours with 10 μM selumetinib prior to confinement. Cell confiner devices were fabricated as described in Le Berre et al (Le Berre et al., 2014). Briefly, a microfabricated dynamic confiner device was attached to a vacuum pump. Polyacrylamide gels were polymerized (60 μL gel solution) on functionalized 10 mm glass coverslips as above. Gel coverslips were then attached to the cell confiner device. To initiate confinement, the confiner device containing the gel coverslip was applied to a 35mm glass bottom dish containing cells. The vacuum pressure was gradually lowered while observing the cells on the microscope with brightfield imaging until the cells were squashed. Images were then acquired on a Zeiss AxioObserver Z1 inverted stand attached to a Yokogawa CSU-W1 spinning disc scan head using a 63x objective

#### Western Blot

Cells were lysed using chilled RIPA buffer on ice. Protein concentration was determined using Bradford reagent and samples were run on a 4-12% Tris/Bis gel (Invitrogen). Protein was then transferred to an Immobilon-P PVDF membrane (Millipore), which was probed using antibodies against the following proteins: phospho-ERK1/2 (Thr202/Tyr204) (Cell Signaling 9101) ERK1/2 (Cell Signaling 9102), phospho-p90RSK (Ser380) (Cell Signaling 9341), RSK1/2/3 (Cell Signaling 9355), phospho-Akt (Ser473) (Cell Signaling 4058), Akt1/2/3 (Santa Cruz sc-81434), Ras (Cell Signaling 3965), E-cadherin (Invitrogen ECCD2) and GAPDH (Thermo Fisher MA5-15738) and then anti-mouse, anti-rabbit and anti-rat HRP-conjugated secondary antibodies (Dako). ECL detection was carried out using the Immobilon Crescendo HRP substrate (Millipore) in an ImageQuant LAS4000 (GE Healthcare).

### Quantification and Statistical Analysis

#### Statistical Analysis

Images were processed and cell shapes measured using FIJI (Schindelin et al., 2012), https://fiji.sc/). Graphs were produced using Prism 7 (GraphPad). Bar charts show mean with error bars showing standard deviation and Box-and-whisker plots use the Tukey method to identify outliers. For live cell imaging, cell shape measurements and gel confinement experiments, graphs show data pooled from at least 3 independent experiments, unless otherwise stated. The number of cells (n) analysed in each condition is indicated in parentheses on plots. For western blotting, at least three independent experiments were carried out and one representative dataset is shown. For AFM, two independent experiments were carried out and one representative dataset is shown. Statistical testing was carried out using Prism 7 (GraphPad). Unless otherwise stated, p values were calculated using the Mann-Whitney two-tailed test. ^∗^p<0.05 ^∗∗^p<0.01 ^∗∗∗^p<0.001. To perform statistical analysis on multiple sets of RT-DC experiments, linear mixed effects models were implemented using the lme4-package in R (R Core Team, 2017; www.R-project.org/.). As fixed effects, we had cell type (control, ras) and cell cycle stage (interphase/mitotis), as random effects, we had random intercepts/slopes for different experimental days. p-values were obtained by likelihood ratio tests of the full model with the effect in question against the model without the effect in question. Further details of the statistical tests used, exact value of n, definition of error bars on graphs can be found in figures and figure legends.

### Data and Code Availability

This study did not generate any unique datasets or code.

## Supplemental Information

Document S1. Figures S1–S3

Document S2. Article plus Supplemental Information
