# Supplementary material for: Oncogenic Signaling Alters Cell Shape and Mechanics to Facilitate Cell Division under Confinement
Source: Dev Cell. 2020 Mar 9;52(5):563–573.e3. doi: 10.1016/j.devcel.2020.01.004 (PMC7063569; doi:10.1016/j.devcel.2020.01.004)
Supplement: Document S1. Figures S1–S3 [file mmc1.pdf]

Developmental Cell, Volume *52*

Supplemental Information

Oncogenic Signaling Alters Cell Shape and Mechanics to Facilitate Cell Division under Confinement

Helen K. Matthews, Sushila Ganguli, Katarzyna Plak, Anna V. Taubenberger, Zaw Win, Max Williamson, Matthieu Piel, Jochen Guck, and Buzz Baum

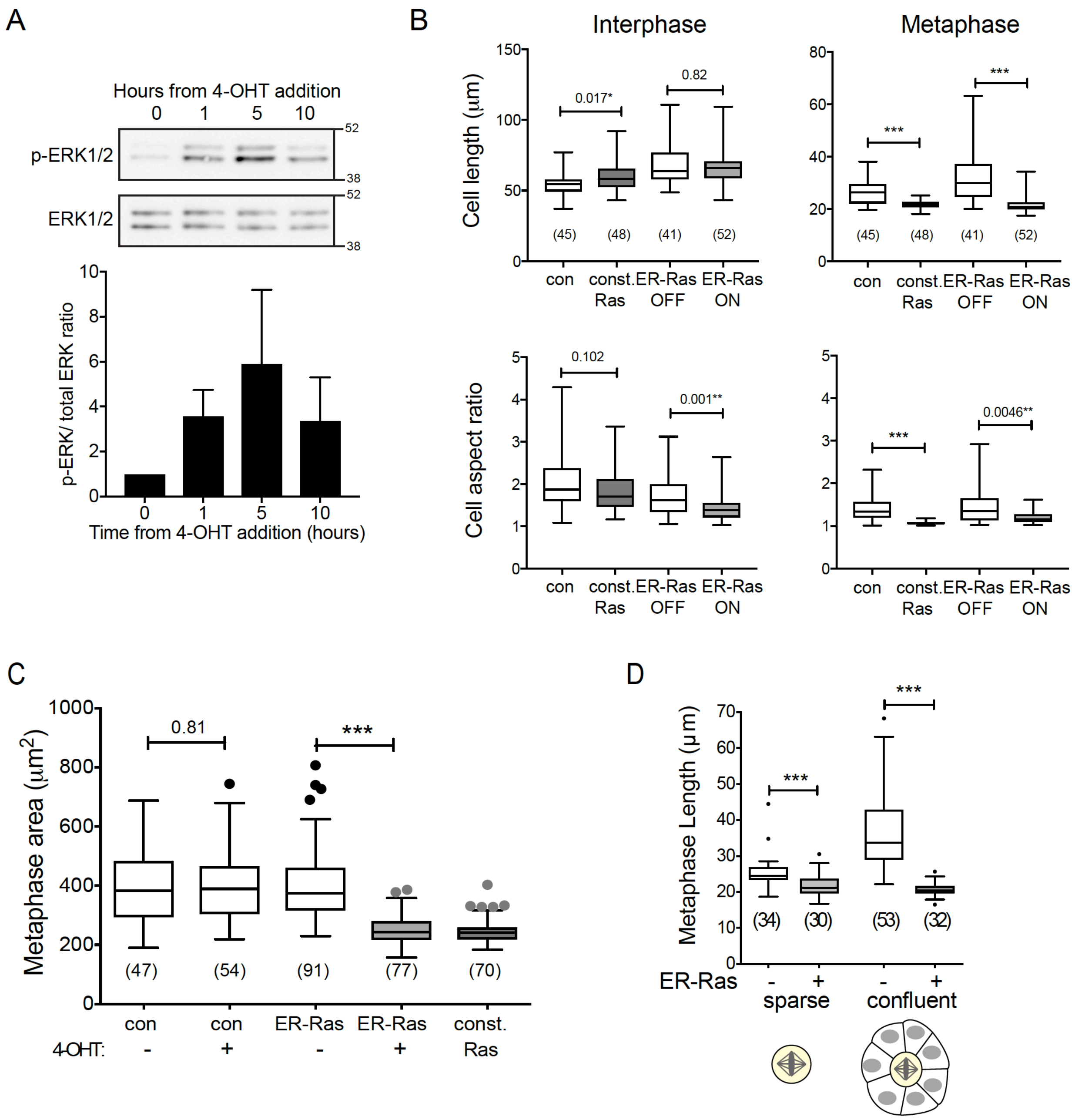

Supplementary Figure S1 (relating to figure 1)

(A) *Ras activation induces downstream ERK phosphorylation.* Western blot showing phospho-ERK and ERK 1/2 protein levels in MCF10A ER-hRas^{V12} cells lysed before 4-OHT addition and after 1, 5 and 10 hours treatment. Graph shows mean phospho-ERK/total ERK ratio quantified from 3 independent blots.

(B) *Ras^{V12} alters cell shape in metaphase but not interphase.* Box plots showing cell length and aspect ratios in interphase and metaphase for the conditions shown in Figure 1C.

(C) *Ras^{V12} activation alters metaphase cell area.* Box plot showing metaphase area for MCF10A and MCF10A ER-hRas^{V12} with 5 -15 hours of ethanol (-) or 4-OH-tamoxifen (+) treatment and for constitutive MCF10A+hRas^{v12} cells. Cells were imaged every 5 minutes for 15 hours using phase contrast microscopy and the shapes recorded for every metaphase (5 minutes before anaphase elongation or furrowing).

(D) *The effect of Ras^{V12} activation depends on cell confluence.* Box plot showing metaphase length for MCF10A ER-hRas^{V12} cells following 5 - 15 hours ethanol or 4-OH-tamoxifen treatment comparing cells plated in sparse (defined as single cells or cells with <2 neighbours) or confluent (cells surrounded by other cells on all sides) conditions.

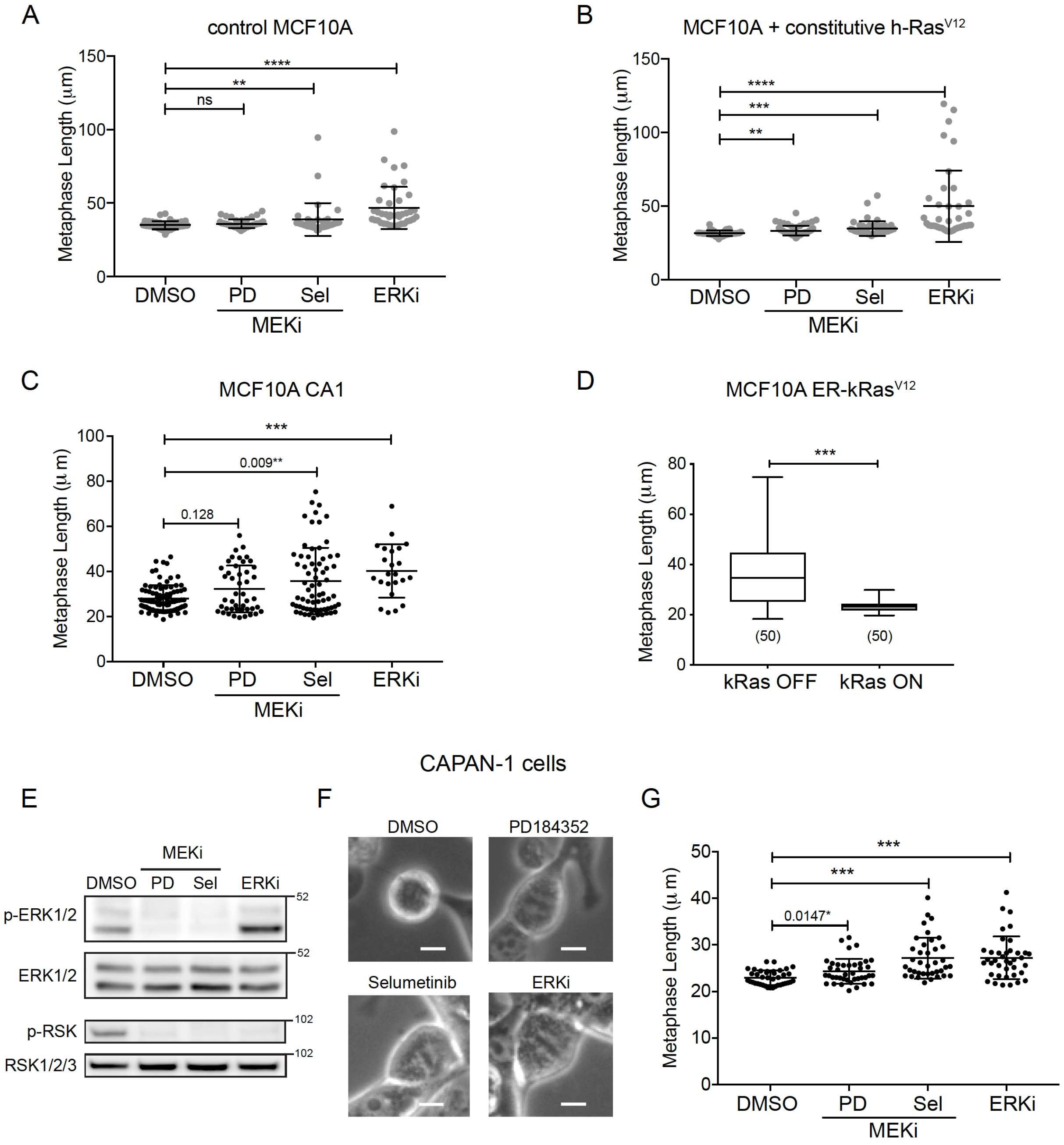

Supplementary Figure S2 (relating to figure 1)

(A-C) *MEK and ERK inhibition alters mitotic shape in normal MCF10A cells, constitutive Ras-expressing cells and h-Ras transformed MCF10A-CA1 cells*. Plot of metaphase length for MCF10A cells (A) constitutive MCF10A+hRas^{v12} cells (B) and MCF10A-CA1 cells (C) following up to 15 hours treatment with DMSO or 2 µM PD 184352, 10 µM Selumetinib or 10 µM GDC-0994. Measurements were taken from time-lapse brightfield images at the frame (5 mins) before anaphase.

(D) *kRas^{V12} affects metaphase shape in a similar way to hRas^{V12}*. Box plot of metaphase cell length (Feret) for MCF10A ER-kRas^{V12} with 5 -15 hours of ethanol or 4-OH-tamoxifen treatment.

(E-G) *MEK and ERK inhibition alters mitotic shape in a kRas-transformed cancer cell line*. Western blot showing ERK and RSK phosphorylation in Capan-1 cells following 5 hours of treatment with 2 µM PD 184352, 10 µM Selumetinib or 10 µM GDC-0994 (E). Representative brightfield images (F) and quantification of metaphase length (G) in Capan-1 cells following 5-15 hours treatment with the same inhibitors. Measurements are taken from time-lapse brightfield images at the frame (5 mins) before anaphase. Scale bars are 10 µm.

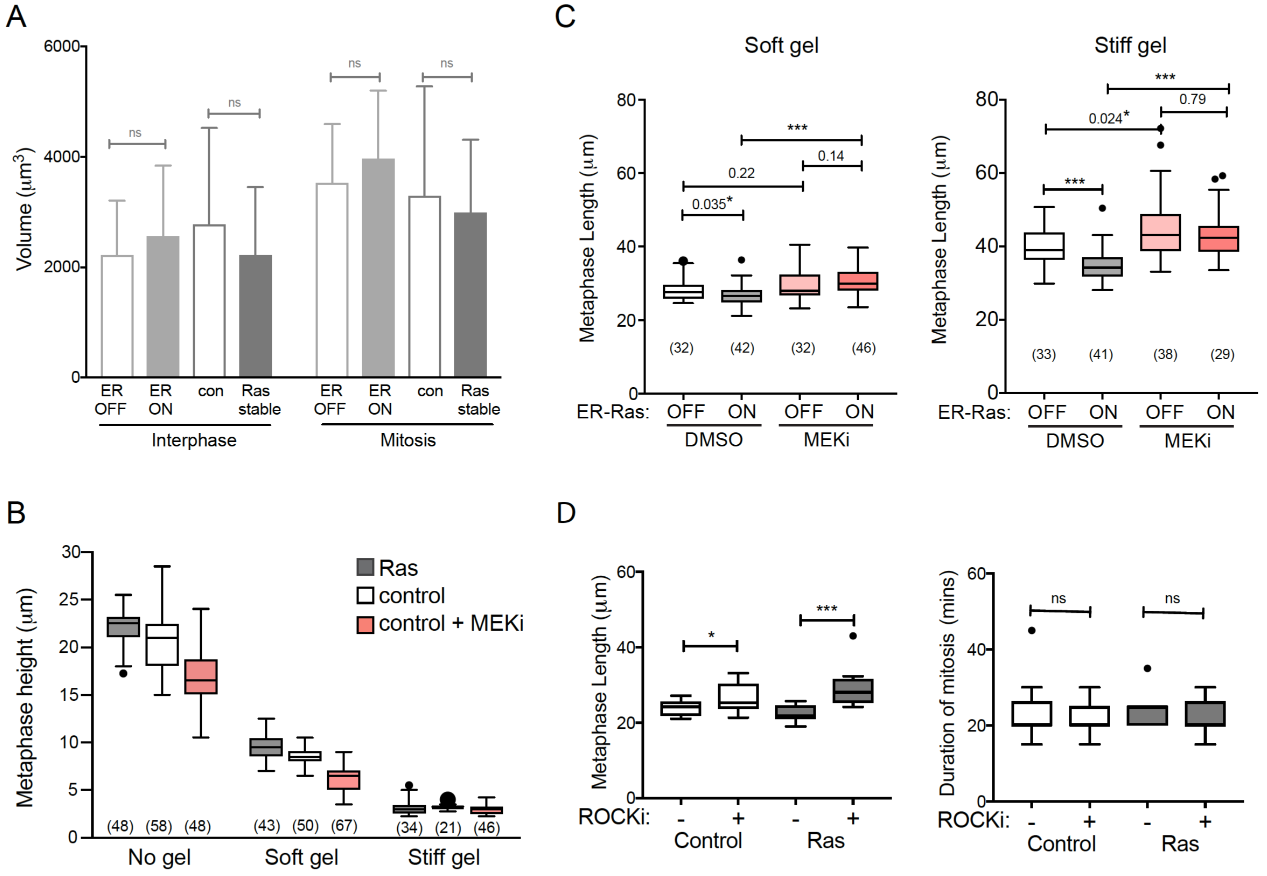

Supplementary Figure S3 (relating to figures 4 & 5)

(A) *Ras-ERK signaling does not alter cell volume.* Mean cell volumes of MCF10A ER-hRas^{V12} after treatment with ethanol or 4-OH-tamoxifen for 15 hours, control MCF10A cells and MCF10A+hRas^{v12} cells in interphase and mitosis. Mitotic cells were synchronized with a 15-hour treatment with 5 µM STLC and harvested by mitotic shake-off. Interphase represents a non-synchronised population harvested using trypsin-EDTA. Volume was measured using a Coulter Counter (Beckman Multisizer 4) for 10,000 cells per condition.

(B) *Ras-ERK signaling alters cell height in unconfined and gel-confined cells.* Box plot showing measurement of maximum height of metaphase cells for MCF10A, MCF10A+ hRas^{v12} and MCF10A treated with 10 µM Selumetinib (MEKi). Measurements were taken from XZ projections (as shown in figure 4C) from the frame before anaphase (3 mins) of confocal timelapse movies. Cells were labeled with 100 nM SiR-Actin to visualize cell borders.

(C) *Short-term RasV^{12} induction enhances the ability of cells to round up under gel confinement.* Box plots showing the metaphase length for MCF10A ER-hRas^{V12} after treatment with ethanol (OFF) or 4-OH-tamoxifen (ON) and DMSO or 2 µM PD 184352 (MEKi) for 5-15 hours, confined under a soft (5 kPa) or stiff (30 kPa) gel.

(D) *ROCK inhibition limits mitotic rounding but does not affect the timing of mitosis in unconfined conditions.* Box plot showing metaphase length and duration of mitosis (time from nuclear envelope breakdown to anaphase) for MCF10A cells and constitutive MCF10A+hRas^{v12} cells following up to 15 hours treatment with or without 25 µM Y-27632. n = 20 cells per condition pooled from 2 independent experiments.
